# Altered Expression of Fibrosis Genes in Capsules of Failed Ahmed Glaucoma Valve Implants

**DOI:** 10.1371/journal.pone.0122409

**Published:** 2015-04-16

**Authors:** Alka Mahale, Maha W. Othman, Sami Al Shahwan, Ibrahim Al Jadaan, Ohood Owaydha, Zahid Khan, Deepak P. Edward

**Affiliations:** 1 King Khaled Eye Specialist Hospital, Riyadh, Kingdom of Saudi Arabia; 2 Genome Research Chair, Department of Biochemistry, College of Science, King Saud University, Riyadh, Kingdom of Saudi Arabia; 3 Wilmer Eye Institute, Johns Hopkins University School of Medicine, Baltimore, Maryland, United States of America; Boston University Goldman School of Dental Medicine, UNITED STATES

## Abstract

**Purpose:**

Ahmed glaucoma valve (AGV) implant is an aqueous shunt device used to control intraocular pressure in glaucoma. Implant failure results from impervious encapsulation of the shunt plate causing increased hydraulic resistance and raised intraocular pressure. We hypothesized that deregulation of fibrosis pathway contributes to capsular resistance. We tested this by studying fibrosis related gene expression in failed AGV implants.

**Methods:**

Differential gene expression was examined in failed AGV capsules and compared to normal control tenon. Following total RNA extraction, 84 key genes in fibrosis pathway were examined by real-time PCR using RT^2^ Profiler PCR Array. Relative gene expression was calculated using ΔΔC_t_ method. Gene specific TaqMan assays were used to validate select genes with ≥2 fold differential expression in the array expression profile.

**Results:**

We observed differential expression in several genes in the fibrosis pathway. Almost half (39/84) of examined genes showed ≥2 fold differential expression in majority of capsules examined on the array. TaqMan assays for select genes including CCN2 (CTGF), THBS1, SERPINE1, THBS2, COL3A1, MMP3, and IL1A in an increased validation sample set showed significant changes in expression (p value from <0.001 to 0.022) at a high frequency in concurrence with our array results.

**Conclusions:**

Pathway-focused analyses identified candidate genes with altered expression providing molecular evidence for deregulation of the fibrosis pathway in AGV failure.

## Introduction

Glaucoma drainage devices are commonly used to lower intraocular pressure in patients unresponsive to maximal medical therapy or failed trabeculectomy [1,2]. Several non-valved and valved varieties are available for clinical use and include Molteno (Molteno Ophthalmic Ltd, New Zealand), Baerveldt (Advanced Medical Optics, USA), Ahmed (New World Medical, USA) and Krupin implants (Hood Laboratories, USA). The Ahmed glaucoma valve (AGV), which is predominantly used at our center, is also the most commonly used valved device along with Baerveldt implant [3]. However, success of this device in lowering intraocular pressure (IOP) declines over time with a failure rate of about 10% per year, thus leading to only 50% functional drainage devices at five years [4,5].

The success of glaucoma shunt surgery is largely dependent on the postoperative healing response and formation of a semi-permeable capsule around the shunt plate allowing aqueous humor drainage and control of IOP [6,7]. The predominant cause of failure resulting in increasing hydraulic resistance is excessive scarring of the encapsulation over the shunt plate, which becomes relatively impermeable [8,9]. The reasons for the excessive fibrotic reaction and capsule impermeability are not fully understood. Apart from implant properties, such as size, shape, surface properties and biomaterial other mechanisms leading to collagen distribution, proliferation and adhesion of fibroblasts have been suggested to contribute to the encapsulation process [10].

Failed AGV capsules have been reported to be macroscopically thicker but histopathologically similar to encapsulated post-trabeculectomy blebs [11]. Additionally, the AGV capsular wall is reported to be macroscopically and histologically split into two layers, with a rough vascularized outer surface and a smooth densely packed fibrous inner surface consisting of compressed collagen fibres and characterized by activated myofibroblasts [12–14]. More recently, bleb imaging with anterior segment OCT showed significantly thinner capsular wall in successful AGV implants compared to unsuccessful implants [8].

Thus, histopathologic and clinical observations of failed and functional capsules have indicated an important role of the fibroblast and its production of extracellular matrix components in pathophysiology of AGV failure [8,12,15,16]. However, the molecular mechanisms that underlie shunt failure due to fibrotic encapsulation have yet to be identified. Fibrosis is a modifiable process and in order to find solutions to regulate the tissue response around shunt capsule, specific molecular targets need to be identified to develop appropriate therapy. Antifibrotics such as Mitomycin C although widely used for the favourable outcomes with trabeculectomy procedures have not been successful in preventing shunt failure [17–20]. Similar efforts to modulate the tissue response with adjunctive amniotic membranes and systemic steroids have also seen limited success [21–23].

We hypothesized that alterations in the molecular events in the fibrosis pathway contributes to increased capsular resistance. Gene expression, especially differential expression data obtained from diseased and control tissues, can provide key information that can help determine genes that may be involved in pathological processes. We therefore examined expression profile of genes involved in the fibrosis pathway in capsules of failed AGV implants. Due to difficulties faced with limited human tissue samples, we designed the study to initially obtain a profile of differential gene expression by targeted array analysis for fibrosis pathway followed by validation of individual changes. To the best of our knowledge such pathway-focused transcript level changes in failed AGV capsules have not been previously investigated.

## Materials and Methods

### Patients

The patients in this prospective study were recruited from the King Khaled Eye specialist hospital, Riyadh, Saudi Arabia. All patients underwent revision of Ahmed glaucoma valve implant (model FP-7; New World Medical, Inc., Rancho Cucamonga, CA) for uncontrolled IOP with maximal tolerated medical therapy. The capsule around the implant, which was smooth-surfaced, thickened, dome-shaped fibrotic tissue, was partially removed during valve revision by glaucoma specialists (DE, SS, IJ, and OO). Immediately after resection, the specimen was transported to the laboratory on ice for further processing. Small pieces of tenon tissue excised during primary valve implantation from eyes without previous filtering procedures were used as a control and were much more limited in size compared to the caspules. The specimens were snap frozen in liquid nitrogen and stored at -80°C until RNA extraction. The following clinical information was documented for each patient- age, gender, time to revision, and IOP.

### Ethics statement

The study followed the principles established in the Declaration of Helsinki and was approved by the Institutional Review Board of King Khaled Eye Specialist Hospital. Written informed consent was obtained from all the study participants and all samples were anonymized to preserve patient confidentiality.

### RNA extraction

RNA was extracted from failed AGV capsules and control tenon tissue by homogenization in TRI Reagent RNA Isolation Reagent (Sigma, St. Louis, USA) and further purified with columns using the RNAqueous-Micro Kit (Ambion, Austin, USA). DNase enzyme digestion was performed to exclude genomic DNA contamination. Challenges were faced for RNA extraction due to the fibrotic nature and small size of the specimens and the above described method was determined to provide optimal yield and quality for analysis. RNA was quantitated and assessed for integrity using NanoDrop 1000 spectrophotometer (Thermo Scientific, Waltham, USA) and Agilent 2100 Bioanalyzer (Agilent Technologies, Santa Clara, USA), respectively. All samples were aliquoted and stored at -80°C before use. Total RNA with 260/280 ratio of 2.0 and RIN of >5.0 was utilized for real-time PCR quantitation by Pathway Signature PCR arrays and RT-PCR TaqMan Gene Expression Assays.

### Real-Time PCR-Based Array Analysis

Relative expression of genes involved in fibrosis was compared between capsules of failed AGV implants and normal control tenon tissues. We utilized Pathway Signature PCR Arrays (SABiosciences Corp., Frederick, USA) to analyse gene expression and determine pathway activity with a single real-time PCR experiment for each specimen. Real-time PCR quantitation was performed on a ViiA7 system (Applied Biosystems, Foster City, USA) using the Human Fibrosis RT^2^ Profiler PCR Arrays (SABiosciences Corp.) consisting of a panel of 84 key genes involved in fibrosis. The first strand cDNA was synthesized with 100 ng of total RNA with the RT^2^ PreAMP cDNA synthesis kit in a 20 μl reaction volume as per manufacturer’s instructions (SABiosciences Corp.). 5 μl of undiluted first strand cDNA was amplified using the RT^2^ PreAMP cDNA Human Fibrosis Primer Mix, which included an initial step at 95°C for 10 min followed by 12 cycles of PCR at 95°C for 15 sec and 60°C for 2 min using DNA Engine Dyad Peltier Thermal cycler (MJ research, Waltham, USA). Samples were then diluted as per supplier’s instructions in RT^2^ SYBR Green qPCR master mix (SABiosciences Corp.) and pipetted into 96-well PCR array plates to evaluate the expression of fibrosis genes. Raw data from the real-time PCR was exported and analysed using the integrated web-based automated software for RT^2^ Profiler PCR Array Data Analysis available through SABiosciences. Raw data sets were uploaded using the PCR array data analysis template available at http://www.sabiosciences.com/pcr/arrayanalysis.php and the quality testing performed using the integrated web-based automated software. The software identified β-actin as the most stably expressed housekeeping gene for data normalization. Relative gene expression was calculated by comparative threshold cycle (ΔΔC_t_) method from uploaded raw threshold cycle data using average cycle threshold value of tenon samples as reference. Thus, fold changes were expressed as the difference in average gene expression of control tenons compared individually with each of the seven capsules. A two fold or greater change in expression was considered significant [24–25].

### TaqMan Gene Expression Assays

Representative genes with ≥2 fold differential expression identified in the fibrosis array were validated by real-time PCR TaqMan gene expression assays (Applied Biosystems, Foster City, USA). Gene specific TaqMan primer assays purchased from Applied Biosystems are indicated in S1 Table. cDNA was prepared with High-Capacity cDNA Reverse Transcription Kits (Applied Biosystems) according to the kit protocol starting with 100–200 ng of total RNA. Real-time PCR was performed on the ViiA7 system in a 96-well format. Each sample was measured in triplicate using the TaqMan Gene Expression Master Mix (Applied Biosystems). The cycling program was set as 50°C for 2 mins, 95°C for 10 mins followed by 40 cycles at 95°C for 15 secs and 60°C for 1 min. The average cycle threshold value for housekeeping gene β-actin was used to normalize the raw cycle threshold data and calculate ΔC_t_. The ΔΔC_t_ for each Gene Of Interest (GOI) was calculated by deducting the average ΔC_t_ of GOI in the control tenons samples from the ΔC_t_ of each GOI in the AGV capsules. The relative gene expression was reported as fold-change of each GOI compared to the control calculated using the formula 2^-ΔΔCt^.

### Statistical Methods

SPSS software package version 19 (SPSS Inc., Chicago, USA) was used for statistical analysis. Descriptive statistics were used to report demographic characteristics. The array experiment was set up in single runs in order to accommodate more biological replicates. Due to the small sample size and low statistical power for our array experiment only simple fold-change criteria of ≥2 fold change was used as cut off to identify significant gene changes. Non parametric Spearman’s rank correlation was used to correlate different genes among themselves and with revision time. Fold change for each GOI from the TaqMan Gene Expression Assay results were used for comparison. Significance of differential gene expression for individual genes examined by TaqMan Gene Expression Assay was calculated by non-parametric Mann-Whitney U test using mean rank of normalized ΔC_t_ values for control tenon versus capsules. Significance level was set at 5% (*p* < 0.05).

## Results

The clinical data of patients who underwent revision are detailed separately for the Array and TaqMan experiments in Table 1. The median time to excision of failed capsules was about 25 months (range 6 to 156 months) indicating mostly late excision. The definition of late excisions is not clear in the literature but is generally considered to be over 2–6 months [12].

**Table 1:** Demographic data and clinical parameters of patients.

	Array		TaqMan gene expression assay	
	Capsules	Tenon	Capsules	Tenon
**n**	7	2	20	8
**Gender (M/F)**	2/5	1/1	8/12	1/7
**Age, Range in years (Median)**	3–67 (28)	16, 55 (N/A)	3–67 (22)	3–64 (28)
**Time to Revision, months Range (Median)**	12–144 (24)	N/A	6–156 (25.5)	N/A
**Implant model**	FP-7	N/A	FP-7	N/A
**Preoperative IOP, mmHg (mean)**	24.6	25	26.15	29.25

### Real-Time PCR array

**Fig 1:**
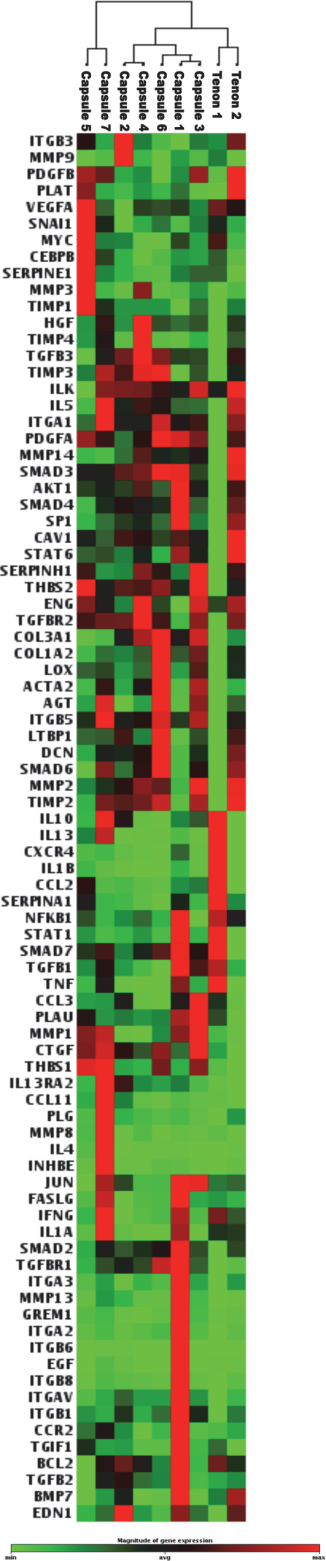
Non-supervised hierarchical clustering of normalized genes examined by PCR analysis.

RNA from seven capsules and two control tenons were examined on the fibrosis array. Using the average cycle threshold value from the two tenon samples as reference, we observed differential expression in several genes in the fibrosis pathway in each of the seven capsules examined by human fibrosis pathway specific profiler PCR array. Non-supervised hierarchical cluster analysis, which pairs samples based on the proximity of their expression profile, clustered the capsules and tenons separately indicating their inherent differences. Variability of the overall gene expression pattern among the excised capsules, especially the distinct pairing of capsule 5 and 7 is indicative of heterogeneity (Fig 1).

**Fig 2:**
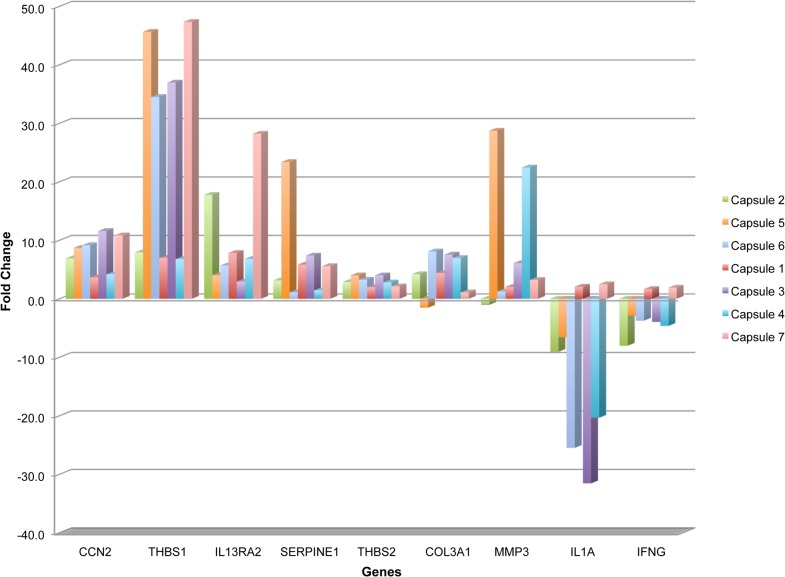
Differential expression of genes identified by Human Fibrosis PCR-Array analysis in failed AGV capsules. Capsules 2, 5 and 6 were from patients below 18 years and capsules 1, 3, 4 and 7 were from patients above 18 years of age. Validated gene changes were deregulated in majority of the capsules.

Forty six percent (39/84) of genes in the fibrosis array showed ≥2 fold differential expression in three or more of the capsules (Table 2). Notably, ≥2 fold upregulation of CCN2 (CTGF) (range: 3.6 to 11.6 fold), THBS1 (range 6.7 to 47.3 fold), IL13RA2 (range 2.9 to 28.2 fold), INHBE (range 2.3 to 195.63 fold) and MMP13 (range 2.3 to 73.1 fold) was found in all 7 capsules. Also, ≥2 fold upregulation of 17 genes including CCL11, THBS2, COL3A1, MMP3, MMP8, and SERPINE1 was seen in ≥57% of excised capsules. Moreover, seven genes including IL1A, IFNG, and BMP7 showed ≥2 fold downregulation in ≥57% of excised capsules. None of the genes showed uniform downregulation in all the capsules. The expression profile of some genes identified in the fibrosis array by our pathway-focused analyses is shown in Fig 2.

**Table 2:** Differential gene expression (≥2 fold) in failed AGV capsules determined by fibrosis array and validated by TaqMan gene expression assays.

Gene	Description	Fibrosis Array	TaqMan validation	Gene Ontology Group(s)
**Upregulated**				
**CCN2**	Connective tissue growth factor (CTGF)	7/7 (100%)	20/20 (100%)	Pro-fibrotic, Growth Factors
**THBS1**	Thrombospondin 1	7/7 (100%)	20/20 (100%)	Signal Transduction:TGFß Superfamily
**IL13RA2**	Interleukin 13 receptor, alpha 2	7/7 (100%)	9/16 (56%)^a^	Pro-fibrotic, Anti-fibrotic, Inflammatory Cytokines and Chemokines
**INHBE**	Inhibin, beta E	7/7	-	Signal Transduction:TGFß Superfamily
**MMP13**	Matrix metallopeptidase 13 (Collagenase 3)	7/7	-	Remodelling enzymes
**CCL11**	Chemokine (C-C motif) ligand 11	6/7	-	Pro-fibrotic, Inflammatory Cytokines and Chemokines
**SERPINE1**	Serpin peptidase inhibitor, clade E (nexin, plasminogen activator inhibitor type 1), member 1	5/7 (71%)	19/20 (95%)	Remodelling enzymes, Epithelial-to-Mesenchymal Transition
**THBS2**	Thrombospondin 2	6/7 (86%)	16/20 (80%)	Signal Transduction:TGFß Superfamily
**COL3A1**	Collagen, type III, alpha 1	5/7 (71%)	15/20 (75%)	ECM components, Epithelial-to-Mesenchymal Transition
**MMP3**	Matrix metallopeptidase 3 (stromelysin 1, progelatinase)	5/7 (71%)	13/20 (65%)	Epithelial-to-Mesenchymal Transition
**AGT**	Angiotensinogen (serpin peptidase inhibitor, clade A, member 8)	5/7	-	Pro-fibrotic, Growth Factors
**HGF**	Hepatocyte growth factor (hepapoietin A; scatter factor)	5/7	-	Anti-fibrotic, Growth Factors
**ITGA2**	Integrin, alpha 2 (CD49B, alpha 2 subunit of VLA-2 receptor)	5/7	-	Cellular adhesion
**ITGB5**	Integrin, beta 5	5/7	-	Cellular adhesion
**LOX**	Lysyl oxidase	5/7	-	Remodelling enzymes
**MMP1**	Matrix metallopeptidase 1 (interstitial collagenase)	5/7	-	Remodelling enzymes
**MMP8**	Matrix metallopeptidase 8 (neutrophil collagenase)	5/7	-	Remodelling enzymes
**ACTA2**	Actin, alpha 2, smooth muscle, aorta	4/7	-	Pro-fibrotic
**COL1A2**	Collagen, type I, alpha 2	4/7	-	ECM components, Epithelial-to-Mesenchymal Transition
**GREM1**	Gremlin 1, DAN family BMP antagonist	4/7	-	Signal Transduction:TGFß Superfamily
**TIMP3**	TIMP metallopeptidase inhibitor 3	4/7	-	Remodelling enzymes
**TIMP4**	TIMP metallopeptidase inhibitor 4	4/7	-	Remodelling enzymes
**Downregulated**				
**IL1A**	Interleukin 1, alpha	5/7 (71%)	13/15 (87%)^a^	Inflammatory Cytokines and Chemokines
**IFNG**	Interferon, gamma	5/7 (71%)	4/15 (27%)^a^	Anti-fibrotic, Inflammatory Cytokines and Chemokines
**BMP7**	Bone morphogenetic protein 7	5/7	-	Anti-fibrotic, Signal Transduction:TGFß Superfamily, Epithelial-to-Mesenchymal Transition
**SERPINA1**	Serpin peptidase inhibitor, clade A (alpha-1 antiproteinase, antitrypsin), member 1	5/7	-	Remodelling enzymes
**CXCR4**	Chemokine (C-X-C motif) receptor 4	4/7	-	Inflammatory Cytokines and Chemokines
**IL13**	Interleukin 13	4/7	-	Inflammatory Cytokines and Chemokines
**IL1B**	Interleukin 1, beta	4/7	-	Inflammatory Cytokines and Chemokines, Proinflammatory cytokine production

^a^Due to sample limitation and poor amplification analysis was only possible in fewer capsules as indicated.

### Validation by TaqMan assay

**Fig 3:**
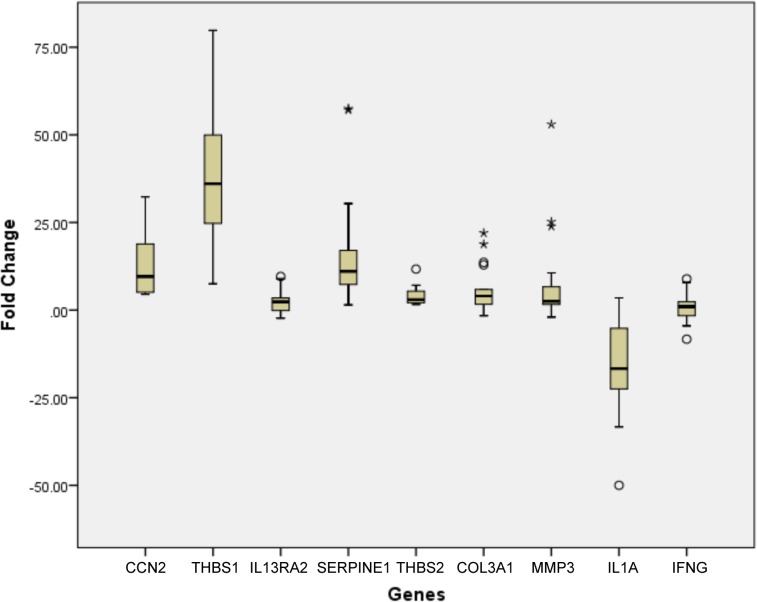
Validation of differential gene expression by TaqMan Gene expression assays. The differential gene expression was statistically significant (p value from <0.001 to 0.022) by non-parametric Mann-Whitney U test except for IFNG and IL13RA2.

Nine representative genes identified to be altered ≥2 fold in the array were further validated by TaqMan gene expression assays in a total of 20 capsules and 8 tenons (Table 2). CCN2 and THBS1 were significantly overexpressed (p<0.001) in all the analysed samples and showed 100% concordance with the array results. Additionally, TaqMan assays for SERPINE1, THBS2, COL3A1, MMP3 and IL1A also showed high concordance with the array results (Table 2). Such concordance between the array and TaqMan results was not seen for IL13RA2 and IFNG. Of the 16 samples analysed for IL13RA2 by TaqMan real-time PCR, nine (56%) showed overexpression compared to 7/7 (100%) overexpression determined by human fibrosis PCR array. Similarly, IFNG was downregulated in a lower proportion 4/15 (27%) by TaqMan assay compared to 5/7 (71%) determined using PCR array (Table 2). The box plot in Fig 3 shows the range of fold changes in the capsules relative to control tenons in each of the validated genes (Fig 3). The differential expression observed during validation for CCN2, THBS1, SERPINE1, THBS2, COL3A1, MMP3 and IL1A showed significant changes (p value from <0.001 to 0.022) at a high frequency in TaqMan assays concurrent with our array results.

We correlated the number of differentially expressed genes (≥2 fold) identified on human fibrosis array panel with age, time to revision and IOP in each of the excised capsules. Spearman’s correlation did not show statistical significance between the number of altered genes identified in the array panel for each capsule and age, revision time or IOP (Table 3).

**Table 3:** Correlation of number of altered genes (≥2 fold) in fibrosis array with age, time to revision and IOP.

	Excised Capsules						
	#2	#5	#6	#1	#3	#4	#7
**No. of altered genes**	34	34	39	42	37	42	39
**Age (yrs)**	3	18	10	67	67	31	28
**Revision Time (months)**	12	12	32	24	24	20	144
**IOP (mm Hg)**	31	33	41	10	20	22	15
** **	No of altered genes vs Age	No of altered genes vs Revision Time		No of altered genes vs IOP		Revision Time vs IOP	
**Spearman’s correlation coefficient**	0.565	0.495		-0.496		-0.346	
**p value**	0.186	0.258		0.258		0.448	

## Discussion

Despite advancements in new glaucoma drainage devices and surgical techniques, fibrosis of the bleb capsule remains the main cause of suboptimal pressure control and failure of drainage procedures [26]. While there is significant research focus on design and other modifications of the device to prevent fibrotic encapsulation, information regarding genes driving the aberrant fibrosis is still lacking. To the best of our knowledge this is the first study using a pathway based gene expression of fibrosis related genes and AGV failure. The available literature is scant and the few concerned studies have investigated histological changes in encapsulation related valve failure. Most of the studies are based on small sample sets (eg, n = 1, 3, 5, 6, 9, 10) and also not always uniform for the implants used [11–13,27–30]. Nevertheless, these studies have provided valuable histopathological insight. Glaucoma is a highly heterogenous group of diseases and in current practice drainage devices are mainly used in refractive cases, furthermore capsular scarring has a multifactorial aetiology [12]. Given these challenges in addition to issues in obtaining sufficient quantity and quality of RNA we have been able to study a relatively larger sample set uniformly for the Ahmed implant than has been investigated before.

Our study revealed differential expression of a large number of genes in the fibrosis pathway, many of them were commonly altered in the failed AGV capsules. Concordance of our array data with highly specific TaqMan assays for several genes validated and suggested the reliability of array findings. The pathway-focused analyses with the fibrosis array together with TaqMan validation experiments identified candidate genes that were up and down regulated, thus providing molecular evidence of alterations in the fibrosis pathway in AGV failure.

In our study, activation of the TGFβ signalling, a known driver of fibrosis was manifest in the deregulation of several molecules and downstream targets in the TGFβ signal transduction cascade (namely GREM1, INHBE, THBS1, THBS2). Although mRNA overexpression of TGFβ2 and TGFBR1 receptor was only detected in 43% of cases, upregulation of several downstream mediators of TGFβ pathway such as CCN2, THBS1 were observed at a very high frequency. In concurrence with above results, TGFβ pathway antagonist (BMP7) was downregulated in majority of the failed capsules lending further support to dysregulation of this pathway. Our findings of differential expression of several members of the TGFβ pathway in majority of cases are in line with studies that demonstrated the role of TGFβ in scarring and fibrosis following glaucoma surgeries [21,31].

Matricellular proteins CCN2 and THBS found altered at high frequency in our study are important modulators of fibrosis and also implicated in glaucoma as causing increased ECM deposition in the trabecular meshwork [32]. Elevated levels of CCN2 and THBS have been reported in aqueous humor [33–35] as well as in the trabecular meshwork of glaucoma patients [36,37] and in other fibrotic conditions [38–39]. Exogenous expression of CCN2 causes glaucoma in mice by modifying the actin cytoskeleton of the trabecular meshwork [40]. Increased CCN2 production is reported in rabbit model of glaucoma filtration surgery [41]. Inhibition of TGFβ and CCN2 in animal models has been shown to counter the scarring and prolong bleb survival indicating these molecules as potential therapeutic targets [42–44]. Considering the evidence from this study and others we believe that these molecules may play a key role in contributing to the increased hydraulic resistance of the capsule.

Notably, no differences were detected in the transcript levels of 11 molecules in the array panel including known drivers of the fibrotic process (TGFβ1, PDGFA and B) between failed capsules and controls. Nonetheless, absence of transcript deregulation for these molecules in our study does not rule out presence of higher protein levels resulting in activation of fibrotic pathway in the AGV capsules. Evidence pertinent to this is that protein levels of TGFβ_2_ receptor (TGFBR2), for which no transcript level differences were detected in our study, has been reported to be elevated in scarred blebs as compared to normal conjunctiva [45]. Additionally, a potential role of the glaucomatous aqueous humor, which is known to be a source for growth factors and proinflammatory substances has been discussed in literature and it has been shown to influence thickness of the capsule wall [46,47]. Moreover, all the scarred blebs studied were excised several months after the initial surgery envisioning the possibility that different subsets of gene are expressed during various stages of the fibrotic process.

Furthermore, we identified overexpression of IL13RA2 that is previously not described in the development of shunt encapsulation. IL13RA2 receptor commonly overexpressed in our samples by array analysis was reported to induce production of TGFβ and fibrosis [48]. This is an interesting finding as IL13RA2 is a soluble receptor for IL13 and blocking IL13RA2 signalling can be a target for prevention of fibrosis. However, these results need further validation as not only have we seen downregulation of IL13 in our samples, but other studies have reported IL13RA2 as a decoy receptor with anti-fibrotic action [49,50].

Increased expression of myofibroblastic marker ACTA2 (α-SMA) seen in our samples was consistent with earlier histopathological observations in capsules where increased α-SMA expression was noted [12]. The increased expression of ACTA-2 together with expression changes in BMP7, COL1A2, COL3A1, MMP3 and SERPINE1 (PAI-1) was indicative of myofibroblastic activity and transformation [12]. In agreement with this, altered expression of extracellular matrix and cell adhesion molecules observed as increased expression of ECM components (COL1A2, COL3A1), several remodelling enzymes (e.g., LOX, MMPs, SERPINA1, SERPINE1 (PAI-1), TIMPs) and cellular adhesion molecules (ITGA2, ITGB5) provides further evidence for the fibrotic process which together stimulate the deposition of connective tissue elements and scarring in our patients. Although there is a consensus on the fibroblastic activity in these capsules most studies report the lack of significant inflammation [12,16,51]. However, we found deregulation of genes expressing several inflammatory cytokines and chemokines (CCL11 (Eotaxin), IL13, IL1A, IL1B, CXCR4 etc) as well as growth factors (AGT, CCN2, HGF) suggesting the presence of autocrine triggers to sustain the fibrotic response. Table 4 highlights the two key processes implicated by our study and their role in increasing hydraulic resistance.

**Table 4:** Key processes implicated in AGV failure.

Key findings	Indicated by	Role in increasing hydraulic resistance
**TGF β pathway upregulation**	Upregulation of downstream effectors (GREM1, INHBE, CCN2, THBS1, THBS2) and downregulation of pathway antagonist (BMP7)	Profibrotic modulation due to deregulation of matricellular proteins and increased ECM deposition
**Myofibroblastic activity and transformation**	Upregulation of several molecules (ACTA-2, COL1A2, COL3A1, LOX, MMPs, ITGA2 etc)	Deposition of connective tissue elements and scarring due to molecular disturbances in ECM, cell adhesion and remodelling enzymes

The gene expression profiles in our cases were consistent with several gene ontology groups. The wound healing process, which progresses through a sequential and overlapping cascade of events is reflected in our array results with some of the identified molecules being categorized in more than one gene ontology group. This suggests the involvement of these molecules at different stages of the fibrotic process as well as an ongoing crosstalk of various pathological processes, all leading to development and maturation of the scar tissue. Activation of TGFβ signalling, found in our samples is known to be associated with excessive scarring and fibrosis following glaucoma surgeries, hence reiterating its importance as a possible target for improving bleb survival. It needs to be underscored that the driver of fibrosis in glaucoma drainage device failure could be the shunt plate or the glaucomatous aqueous as the accumulating evidence suggests [52,53]. Therefore research aimed at developing effective non-toxic alternatives to prevent fibrosis could be directed at both levels [26,54,55]. More recently newer agents targeting specific growth factors, such as anti-vascular endothelial growth factors (anti-VEGF) and anti-TGF-β2 are being investigated [56–59].

There are limitations in our study. The statistical significance could not be evaluated for the array results due to small number of samples that were examined. Additionally, due to small surgical specimen sizes only limited amounts of RNA could be obtained and genes that needed more template for amplification could not be validated by TaqMan assays. The capsules examined in our study were mostly late excisions and while our study demonstrates that at a late stage there is active TGFβ driven fibrosis, gene expression changes in early stages of bleb failure could not be determined. Additionally, the capsules were exposed to extensive medical therapy for glaucoma and effects of these medications on gene changes in these capsules largely remain unknown. Regardless of these limitations, our findings demonstrate clear alterations in fibrosis related gene pathways in AGV failure and these preliminary findings will help form the basis for systematic future investigations. Additional studies are needed to further validate the identified gene expression changes at the protein level. Increasing the sample size in future studies would give the statistical power to allow for better correlation of the gene changes with clinical parameters such as age, revision time, etc. It would also be interesting to examine the effects of glaucoma medications and previous ophthalmic procedures on gene expression and capsule survival.

In summary this study identified important molecular players in AGV failure and provides a preliminary yet global view of the fibrosis pathway activity via gene expression changes. Our results, which are in concurrence with earlier histological reports, provide further comprehensive evidence for transcript level changes in several molecules and processes implicated in the failure of glaucoma drainage surgeries. Characterizing these dysregulated genes will aid in understanding the complex network underlying the fibrotic pathway and development of appropriate antifibrotic targets to suppress the impervious capsule formation and achieve long-term AGV performance and IOP control.

## Supporting Information

S1 Table List of TaqMan Gene Expression Assay IDs. (DOCX)
