# Supplementary material for: Altered Expression of Fibrosis Genes in Capsules of Failed Ahmed Glaucoma Valve Implants
Source: PLoS One. 2015 Apr 16;10(4):e0122409. doi: 10.1371/journal.pone.0122409 (PMC4399875; doi:10.1371/journal.pone.0122409)
Supplement: S1 Table — (DOCX) [file pone.0122409.s001.docx]

**S1 Table. List of TaqMan® Gene Expression Assay IDs.**

| **Gene** | **TaqMan^®^ Gene Expression Assay ID** |
| --- | --- |
| **CCN2** | Hs01026927_g1 |
| **IL13RA2** | Hs00152924_m1 |
| **THBS1** | Hs00962908_m1 |
| **COL3A1** | Hs00943809_m1 |
| **MMP3** | Hs00968305_m1 |
| **SERPINE1** | Hs01126606_m1 |
| **THBS2** | Hs01568063_m1 |
| **INFG** | Hs00989291_m1 |
| **IL1A** | Hs00174092_m1 |
| **ACTB** | Hs01060665_g1 |
